# Tree mortality in response to typhoon-induced floods and mudslides is determined by tree species, size, and position in a riparian Formosan gum forest in subtropical Taiwan

**DOI:** 10.1371/journal.pone.0190832

**Published:** 2018-01-05

**Authors:** Hsy-Yu Tzeng, Wei Wang, Yen-Hsueh Tseng, Ching-An Chiu, Chu-Chia Kuo, Shang-Te Tsai

**Affiliations:** 1 Department of Forestry, National Chung Hsing University, Taichung, Taiwan; 2 Experimental Forest Management Office, National Chung Hsing University, Taichung, Taiwan; 3 Deptartment of Sustainable Tourism, Graduate Institute of Environmental Resources Management, TransWorld University, Douliou, Yunlin, Taiwan; University of Waikato, NEW ZEALAND

## Abstract

Global warming-induced extreme climatic changes have increased the frequency of severe typhoons bringing heavy rains; this has considerably affected the stability of the forest ecosystems. Since the Taiwan 921 earthquake occurred in 21 September 1999, the mountain geology of the Island of Taiwan has become unstable and typhoon-induced floods and mudslides have changed the topography and geomorphology of the area; this has further affected the stability and functions of the riparian ecosystem. In this study, the vegetation of the unique Aowanda Formosan gum forest in Central Taiwan was monitored for 3 years after the occurrence of floods and mudslides during 2009–2011. Tree growth and survival, effects of floods and mudslides, and factors influencing tree survival were investigated. We hypothesized that (1) the effects of floods on the survival are significantly different for each tree species; (2) tree diameter at breast height (DBH) affects tree survival–i.e., the larger the DBH, the higher the survival rate; and (3) the relative position of trees affects tree survival after disturbances by floods and mudslides–the farther trees are from the river, the higher is their survival rate. Our results showed that after floods and mudslides, the lifespans of the major tree species varied significantly. *Liquidambar formosana* displayed the highest flood tolerance, and the trunks of *Lagerstoemia subcostata* began rooting after disturbances. Multiple regression analysis indicated that factors such as species, DBH, distance from sampled tree to the above boundary of sample plot (far from the riverbank), and distance from the upstream of the river affected the lifespans of trees; the three factors affected each tree species to different degrees. Furthermore, we showed that insect infestation had a critical role in determining tree survival rate. Our 3-year monitoring investigation revealed that severe typhoon-induced floods and mudslides disturbed the riparian vegetation in the Formosan gum forest, replacing the original vegetation and beginning secondary succession. Moreover, flooding provided new habitats for various plants to establish their progeny. By using our results, lifecycles of trees (including death) can be understood in detail, facilitating riparian vegetation engineering in forests severely disturbed by typhoon-induced floods and mudslides.

## Introduction

Typhoons have a crucial role in the forest ecosystem of Taiwan. On average, Taiwan is struck by 3.5 typhoons each year, which are strongly correlated with the reproduction, regeneration, and succession of tree species [1, 2, 3, 4, 5]. In recent years, global warming-induced extreme climatic changes have increased the frequency of extremely heavy rainfalls brought by severe typhoons [6, 7, 8]. These climatic changes have largely affected the stability and functions of forest ecosystems [9, 10].

Since the Taiwan 921 earthquake occurred in 21 September 1999, the mountain geology of the central area has been affected by increased frequencies of extreme torrential rains, mountain collapses, and mudslides. During 2008–2009, the Typhoons Kalmaegi, Sinlaku, and Morakot brought extra-heavy rainfall; typhoon-induced floods and mudslides changed the topography and geomorphology of the area, further influencing the stability and function of the riparian vegetation at medium altitudes. This currently remains a critical issue for forest management in Taiwan [11, 12, 13].

Riparian vegetation is an ecological ecotone, possessing high biodiversity and environmental diversity. Therefore, it requires different management strategies than the general forest environment does. Furthermore, riparian vegetation can be a major habitat of wild animals and a source of coarse wood debris and nutrients in creeks. It affects the microclimate of the creeks and protects water quality [14]. Floods are critical and complex factors influencing riparian vegetation; the frequency, intensity, duration, and season of flood events and amount of mud brought by them affect the composition, structure, and spatial distribution of riparian vegetation [15, 16, 17]. During long-term adaptation and evaluation, riparian species have evolved the ability to escape or adapt to floods. However, the high frequency and severity of floods caused by climatic changes have compromised the stability of riparian ecosystem [11, 17, 18, 19, 20].

**Fig 1:**
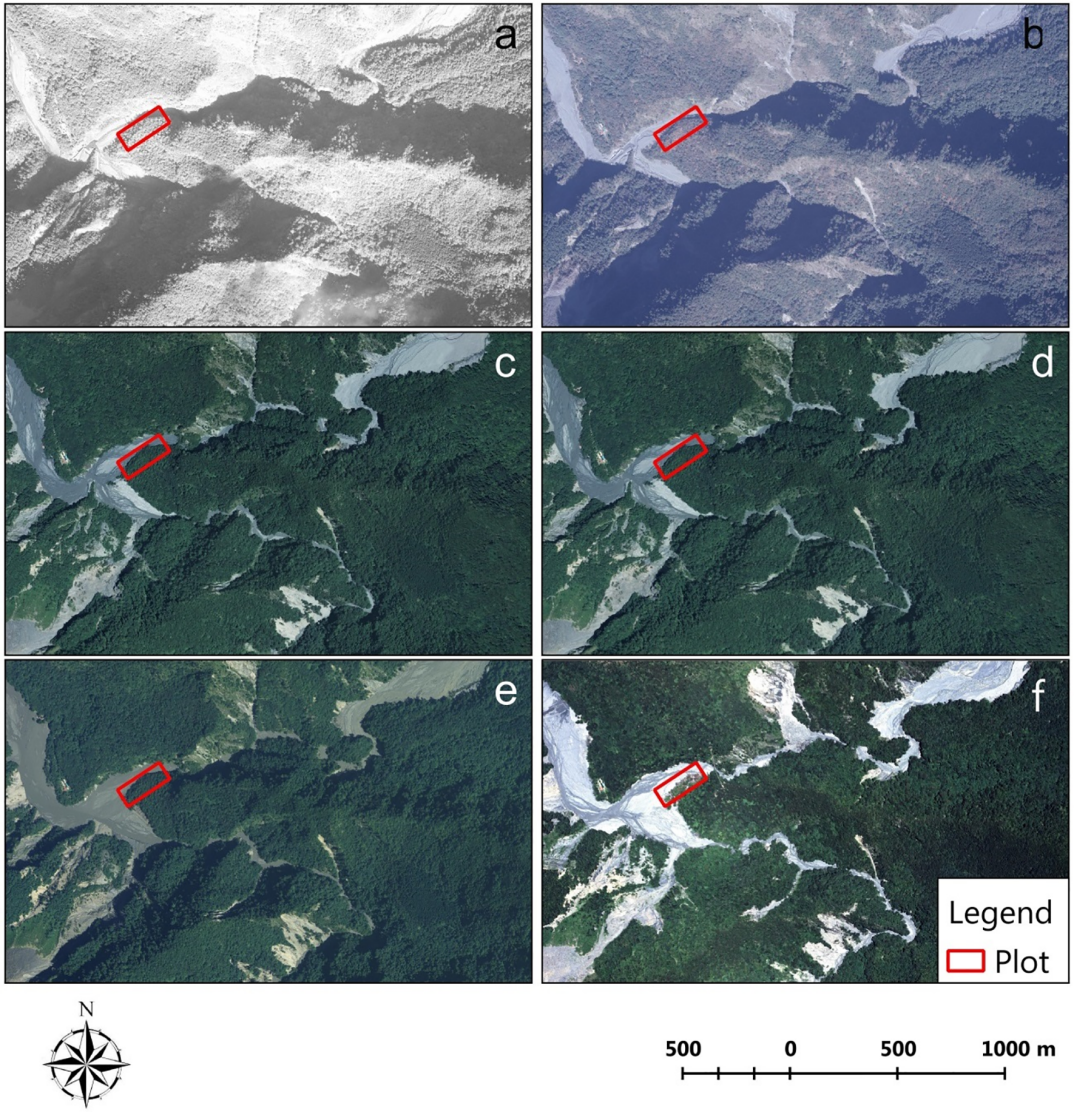
Aerial photographs of the location of the monitored sample Aowanda Formosan gum forest plot over the years: (a) 1996, (b) 1999, (c) 2007, (d) August 2008, (e) September 2008, and (f) 2011.

The Aowanda Formosan gum forest is the most crucial natural landscape in the Aowanda National Forest Recreation Area. The Formosan gum forest is dominant by deciduous *Liquidambar formosana* and this forest type is rare in subtropical Taiwan. Located at the intersection of the south and north Wanda River, these trees constitute a part of riparian vegetation on the higher river terrace [11]. Before the Taiwan 921 earthquake, the average distance from the river level to Formosan gum forest terrace was more than 5 m. However, since the earthquake occurred in Central Taiwan on September 21, 1999, large-area landslides have occurred upstream of the north Wanda River. With the incursion of several typhoons bringing large amount of rainfall during 2008–2009, the riparian vegetation in the Formosan gum forest became overrun by rivers that changed course, and sand and gravel were deposited on the forest floor (Fig 1).

In this study, the plant community and habitat in the Formosan gum forest were monitored for 3 years. The growth and survival of the tree species within the forest were investigated, and the effect of floods and mudslides on tree survival was analyzed. We hypothesized that (1) the effects of floods on survival are significantly different for each tree species; (2) tree diameter at breast height (DBH) affects tree survival, i.e., the larger the DBH, the higher the survival rate; and (3) the relative position of trees affects tree survival after disturbances by floods and mudslides–the farther the trees are from the river, the higher is their survival rate (i.e., trees distributed downstream have higher survival rates because trees located upstream provide protection; trees located closer to the stream have higher mortality rates due to disturbance more by flood.).

## Materials and methods

### Study area

**Fig 2:**
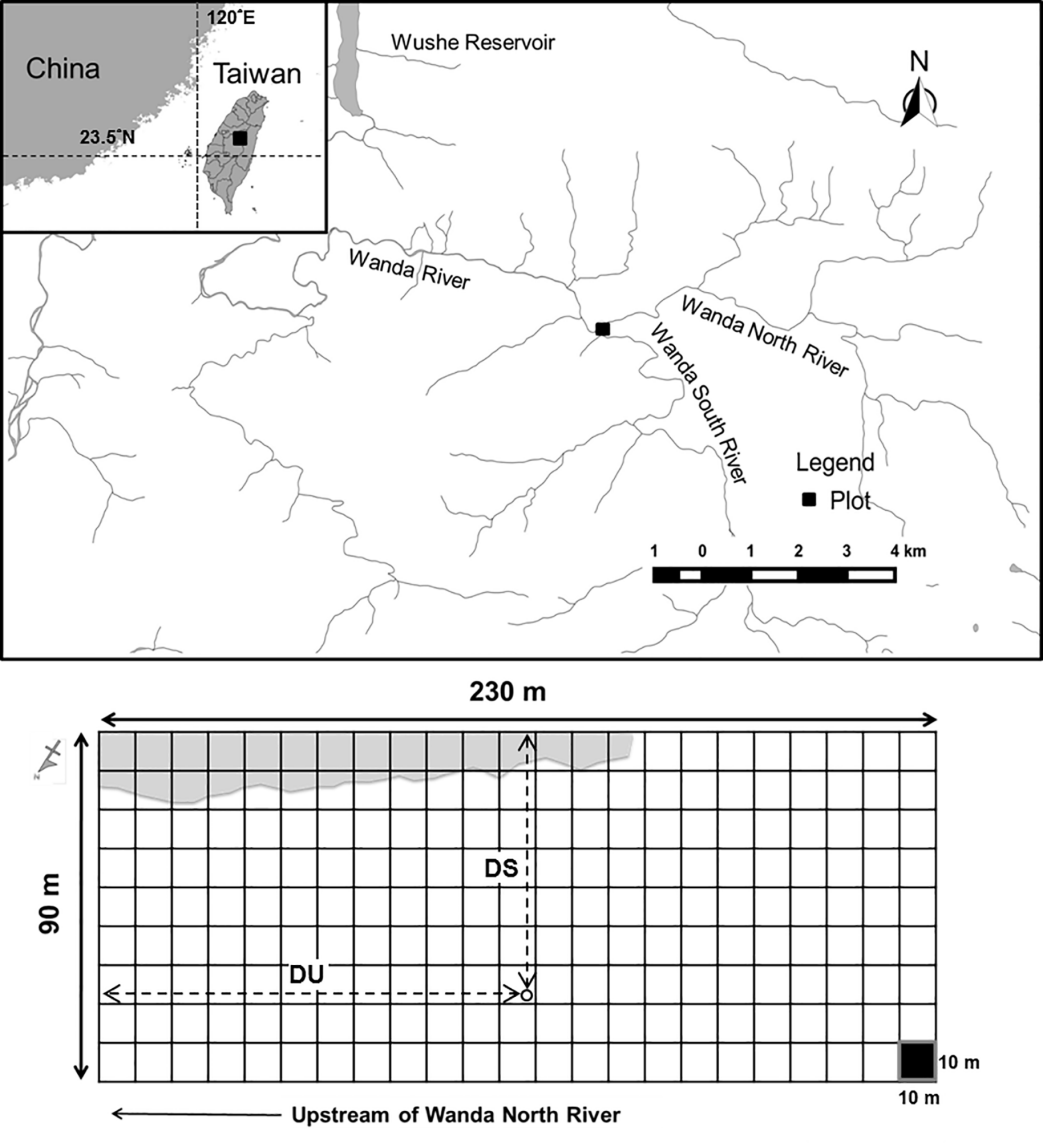
Location (above) of and set up (below) in the monitored sample plot. Gray areas (below) indicate areas free from flood and sand and gravel deposition. DS indicates the distance from sampled tree (circle) to the above boundary of sample plot; DU indicates the distance from sampled tree (circle) to the upstream boundary of sample plot.

The study area is located in the Aowanda Formosan gum forest in the Aowanda National Forest Recreation Area in Central Taiwan, which contains alluvial terrace from the river bed of the south and north Wanda River (Figs 1 and 2). The area, approximately 8 ha, is located 1,240 m above sea level (23°56′45′′N, 121°10′50′′E). The Wanda River is the main tributary, upstream of the Choshui River, the longest drainage area in Taiwan, which has a rapid flow rate and steep riverbanks. The bedrock of Wanda River is mainly composed of the Miocene Lushan formation slate and Quaternary colluvium containing unconsolidated gravel and is distributed mainly on the flat terraces and surfaces of gradual slopes. The colluvium contains gravel from steep slopes and piles of sand at the basal slope [21]. Soil mainly contains gravel mountain soil and humus loams [22]. The vegetation belongs to the *Liquidambar formosana* subtype, *Cinnamomum insularimontanum* type community of the Wanda River drainage area [22].

**Fig 3:**
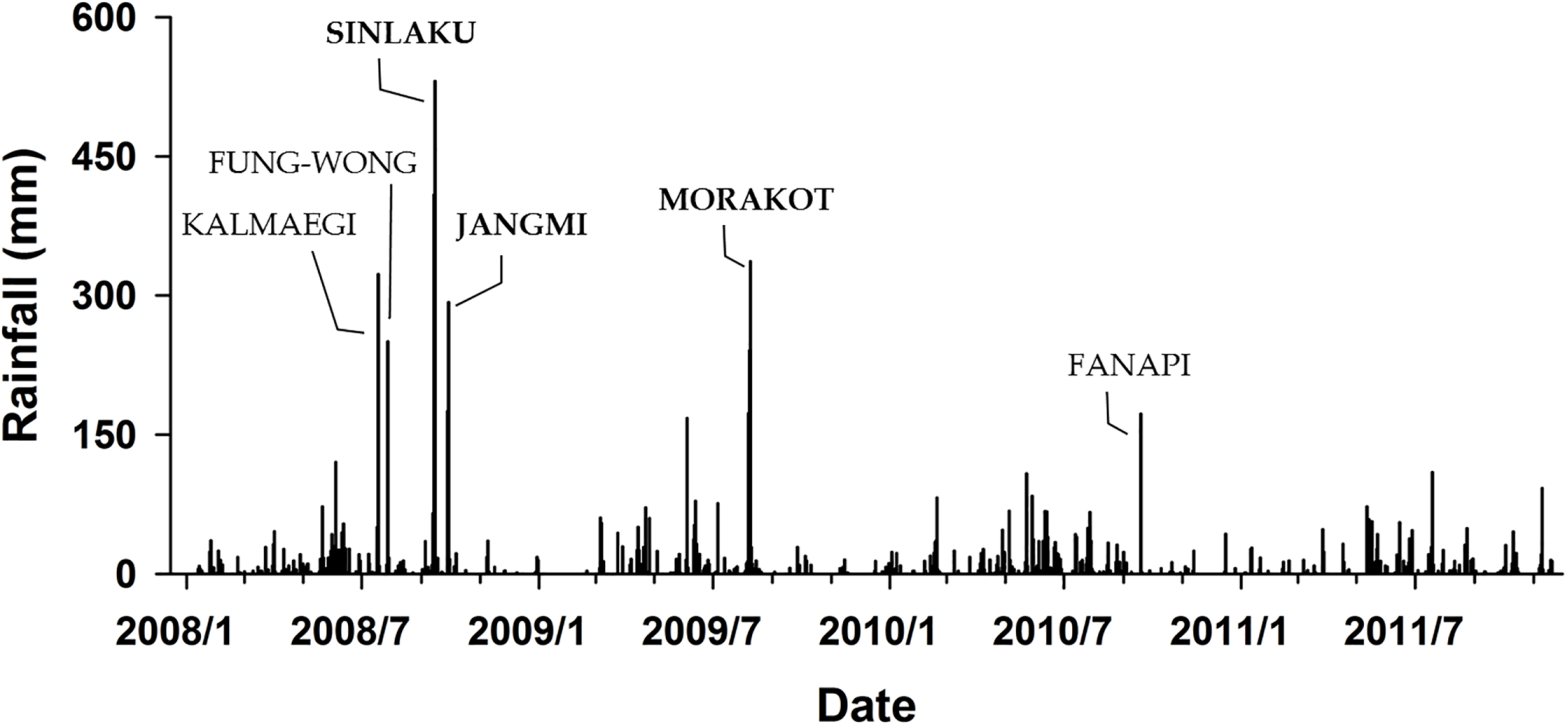
The relationship of daily rainfall changes with typhoons in the monitored sample plot during January 2008 to September 2011 (as per the automatic rainfall measurement station at Aowanda, Central Weather Bureau).

According to the information on rainfall in the study area during 2008–2011 (Fig 3), a heavier daily rainfall occurred during typhoons; the daily rainfall levels were >500 mm during Typhoon Sinlaku in 2008–more than the standard levels for extremely torrential rain (350 mm/24 h), as defined by the Central Weather Bureau [23] during Typhoons Kalmaegi, Fungwong, and Jangmi in 2008 and Typhoon Morakot in 2009, daily rainfall levels were >250 mm–more than the standard levels of torrential rain (200 mm/24 h). Five typhoons infiltrated Taiwan in 2011, namely Aera, Songda, Meari, Muifa, and Nanmadol. Although the number of typhoons was more than that observed 3 years previously, the amount of rainfall had a relatively weaker effect on the Aowanda area. Affected by the simultaneous effects of the northeast monsoon and southwest airflow, Central and Northern Taiwan consecutively encountered heavy rains in October 2011 (Fig 3), and the accumulated amount of rainfall had a major effect on Central Taiwan [23].

### Vegetation and tree survival survey

A long-term monitored plot (100 × 230 m) was established in the Formosan gum forest on the riverbank of the north Wanda River in December 2008 (Fig 2) [11]. The range of the sample area included the area infiltrated by floods, and its vertical axis was parallel to the direction of the river flow. The sample areas were divided into subareas, each sized 10 × 10 m. The survey record illustrated that the plants in the area belonged to 44 families, 69 genera, and 73 species [11]. Groundcover plants such as ferns, grasses, and seedling and juvenile trees were mostly scoured by floods or buried by sand and gravel. The topographies of the north Wanda River and the Formosan gum forest were altered because of frequent heavy rainfalls and mudslides. In the sample area, trees with DBH > 2 cm were classified as part of the tree layer. Trees with DBH >2 cm were measured, and the relative location of the trees within the area was surveyed. And then, the sample trees were identified to species and marked the number on the tag tying on the tree. DBH was >2 cm for 1,201 trees, and the tree layer comprised five dominant species including *Liq*. *formosana*, *Zelkova serrata*, *Ci*. *insularimontanum*, *Schefflera octophylla*, and *Cyclobalanopsis glauca* [11].

The trees in the sample area were resurveyed during 2009–2011 over 12 seasons in total, including spring (March–May), summer (June–August), fall (September–November), and winter (December–February), each year. The records were denoted in 2009 as season 1 (January), 2 (March), 3 (August), and 4 (November); in 2010 as season 5 (January), 6 (March), 7 (July), and 8 (October); and in 2011 as season 9 (January), 10 (April), 11 (August), and 12 (October). In each season, the growth status (survived or withered) of tree species on the monitored plot was surveyed. The “withered tree” was defined to the tree with abundant leaves becoming tawny and dry when we survey at first time. If the tree was marked “withered” at the current survey and it was still “withered” at next survey, which the withered tree was deal with as dead tree. The survival rate of tree species in different seasons was analyzed and compared, and the effects of the tree DBH and location on tree survival rate were investigated. In addition to the monitored survey, vegetation photos were taken on the fixed position for each survey period at the same time. Otherwise, we uploaded the raw data of our study as Supporting information as a “S1 Dataset” file.

### Data analysis

We examined the species properties on the basis of importance values (IV) in each subplot. The IV for each species per subplot was calculated as the sum of relative density (number of individuals of one species/total number of all species individuals counted) and relative dominance (basal area of one species/total basal area of all species). Cluster analyses were then performed on the plant community by using the subplot as a single unit. Similarities between two areas were evaluated using the Bray-Curtis index, and data were linked using the nearest neighbor method. The data were then used to create a dendrogram. These analyses were conducted using the PC-ORD 5.0 software.

Tree population structure indicates the relationship between the age of the tree and its distribution within the tree population and provides information to understand the population regeneration and dynamic for predicting the growth or recession of tree population in the past or future [24]. Because the accurate determination of tree age is difficult, DBH can be used as an indicator. Therefore, the relationship between different diameter groups and the number of the trees within in the group (e.g., size class structure) can be an index for determining tree population dynamics [25]. In this study, the size class structures of the dominant tree species on the plot were investigated to provide information for further discussion regarding population dynamics, forest regeneration, and succession.

Based on the results of the seasonal investigation, the number of the months sample trees survived was calculated (as the number of survival days/30); growth status was indicated using DBH, and position factors were using distance from sampled tree to the above boundary of sample plot (DS) and distance from upstream (DU) (Fig 2). Due to heavy rainfall causing the river from time to time occur several times changed, we use the distance from sampled tree to the above boundary of sample plot to replace the distance from the riverbank. DS was resembled as the reciprocal of the distance from the riverbank in our study. We used the Kruskal-Wallis one-way analysis of variance (ANOVA)–a nonparametric equivalent of ANOVA–to compare the difference in the lifespan (in months) of trees among different species. The following hypothesis was then proposed:

$$\{\begin{array}{c} H_{0}:y=\beta_{0}+\varepsilon \\ H_{1}:y=\beta_{0}+\beta_{1}x_{1}+\beta_{2}x_{2}+\beta_{3}x_{3}+\varepsilon \end{array}$$

In the equation, *y* indicates the lifespan of trees (in months), *x*_1_ indicates DBH, *x*_2_ indicates distance from the upper boundary of the sample plot, and *x*_3_ indicates distance from upstream. We used multiple regression analyses to investigate the effect of DBH, the distance from the upper boundary of the sample plot, and the distance from upstream on the lifespans of trees. Then, we used the Kolmogorov-Smirnov test–a nonparametric test that compares pairs of samples–to compare differences among groups.

## Results

### Vegetation structure

**Fig 4:**
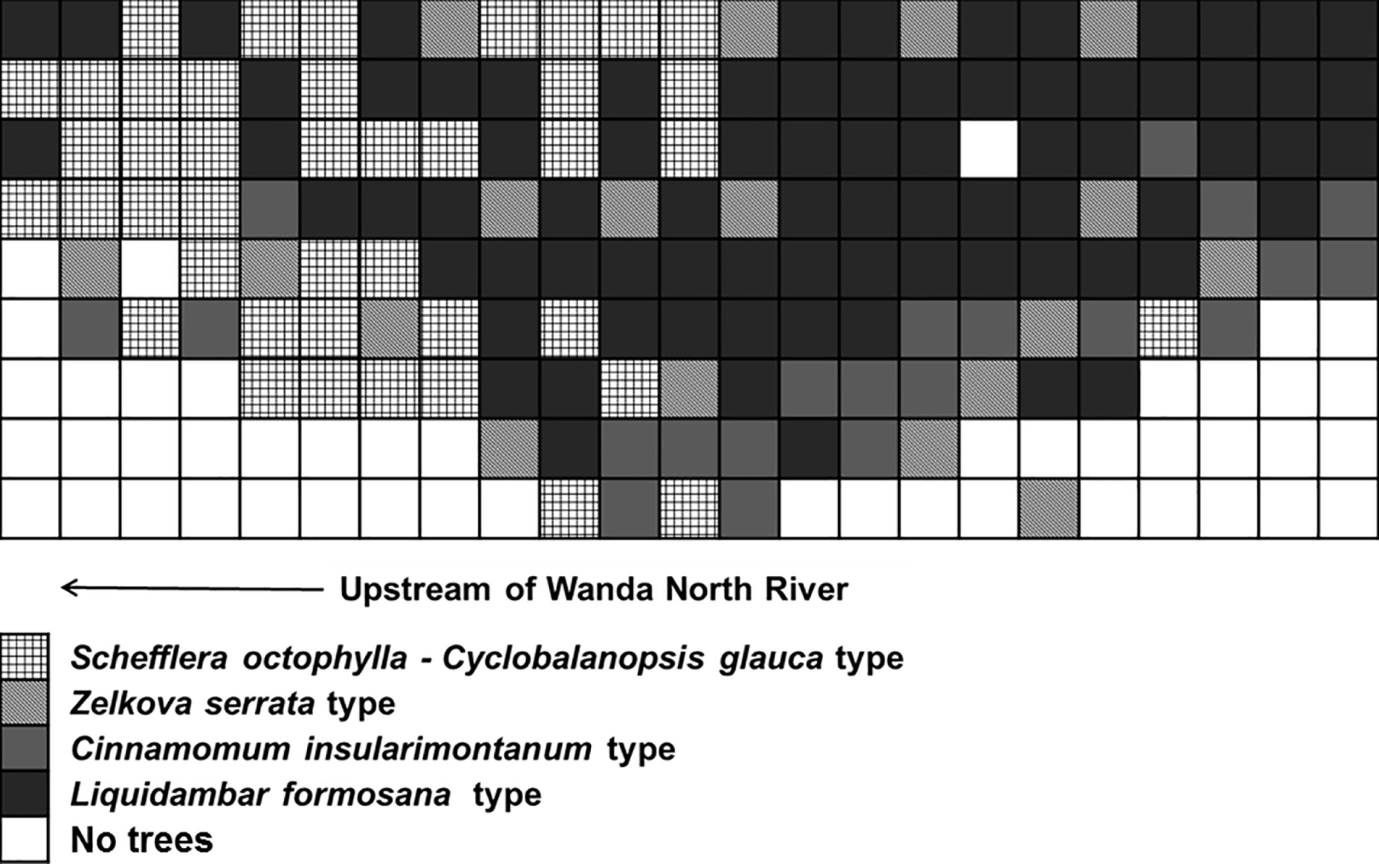
Vegetation distribution in the monitored sample plot.

During 2008–2009, when the Aowanda Formosan gum forest was affected by typhoons with heavy rain, a part of the Formosan gum forest was deposited by at least 5 m of water-carried sand and gravel. Most hardwood trees were of *Liq*. *formosana* (261 trees), followed by *Z*. *serrata* (189 trees), *Ci*. *insularimontanum* (159 trees), *Sc*. *octophylla* (92 trees), and *Cy*. *glauca* (90 trees). These trees comprised the major canopy in the area. Among these, *Liq*. *formosana* and *Z*. *serrata* belonged to the crown canopy, whereas *Ci*. *insularimontanum*, *Sc*. *octophylla*, and *Cy*. *glauca* belonged to the lower canopy. The results of our cluster analysis revealed that the dominant species could be classified into four types: *Sc*. *octophylla*–*Cy*. *glauca*, *Z*. *serrata*, *Ci*. *insularimontanum*, and *Liq*. *formosana* types (Fig 4). Among these, the *Liq*. *formosana* type had dominant distribution in the sample area, whereas the *Sc*. *octophylla–Cy*. *glauca* type, dominant during the middle and late stages of forest succession, was distributed in the more elevated areas. The *Z*. *serrata* type was widely distributed within the sample area and tended to be more dominant along the outer rim of the sample area. Finally, the *Ci*. *insularimontanum* type was mainly concentrated along the margin of the area.

**Fig 5:**
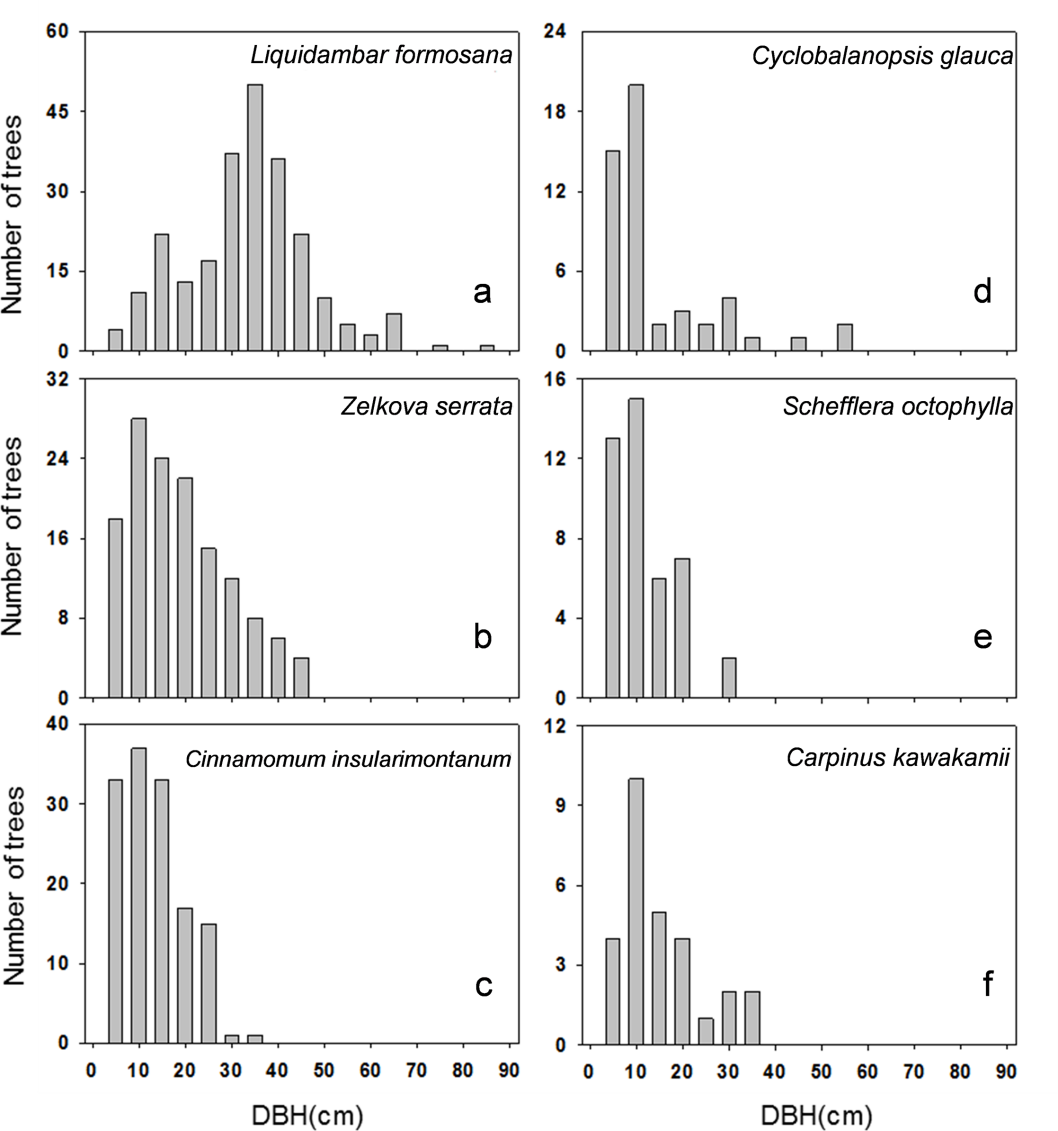
Distribution of size classes of the major tree species in the monitored sample plot.

**Fig 6:**
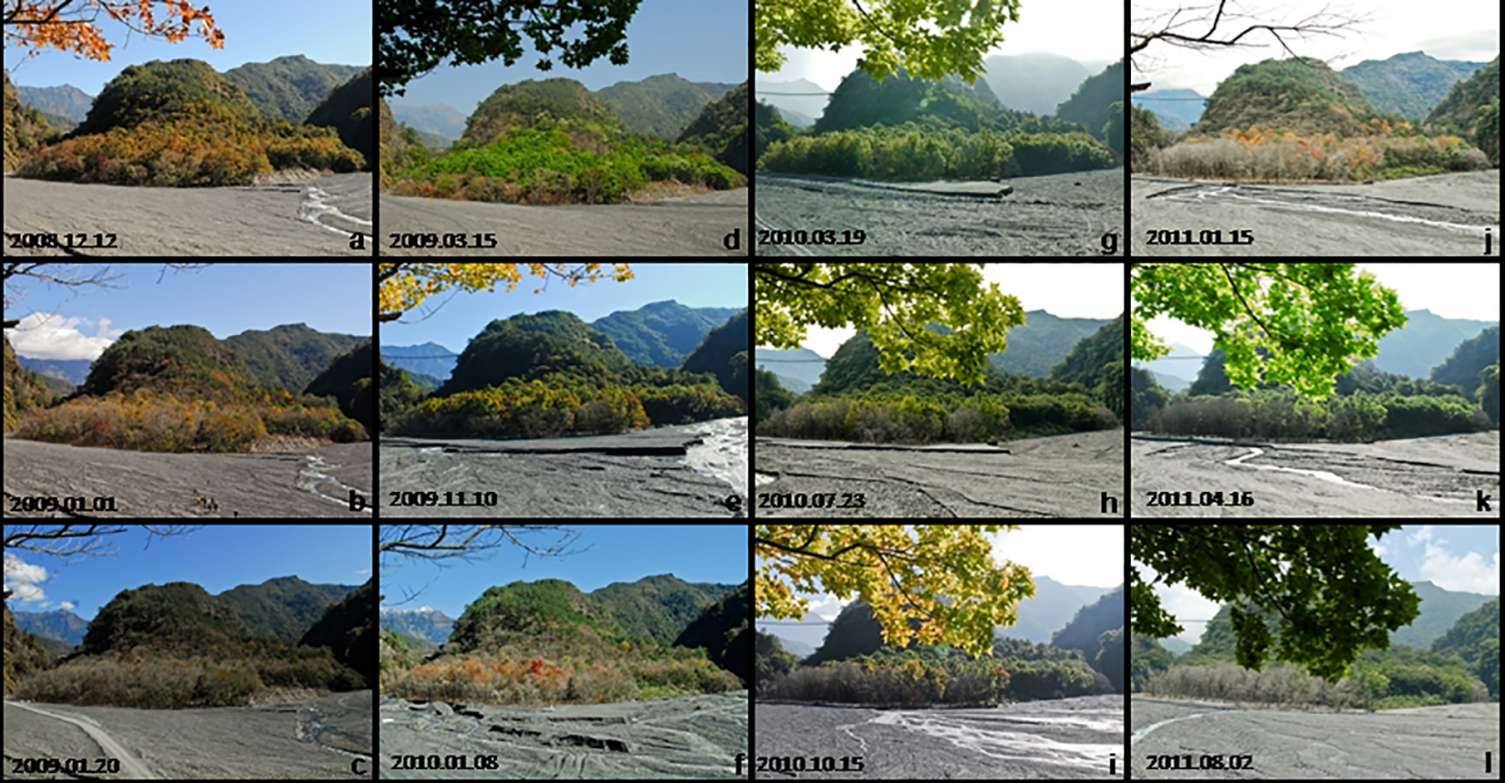
Seasonal changes of vegetation physiognomy after disturbances by floods and mudslides in the monitored sample plot.

**Fig 7:**
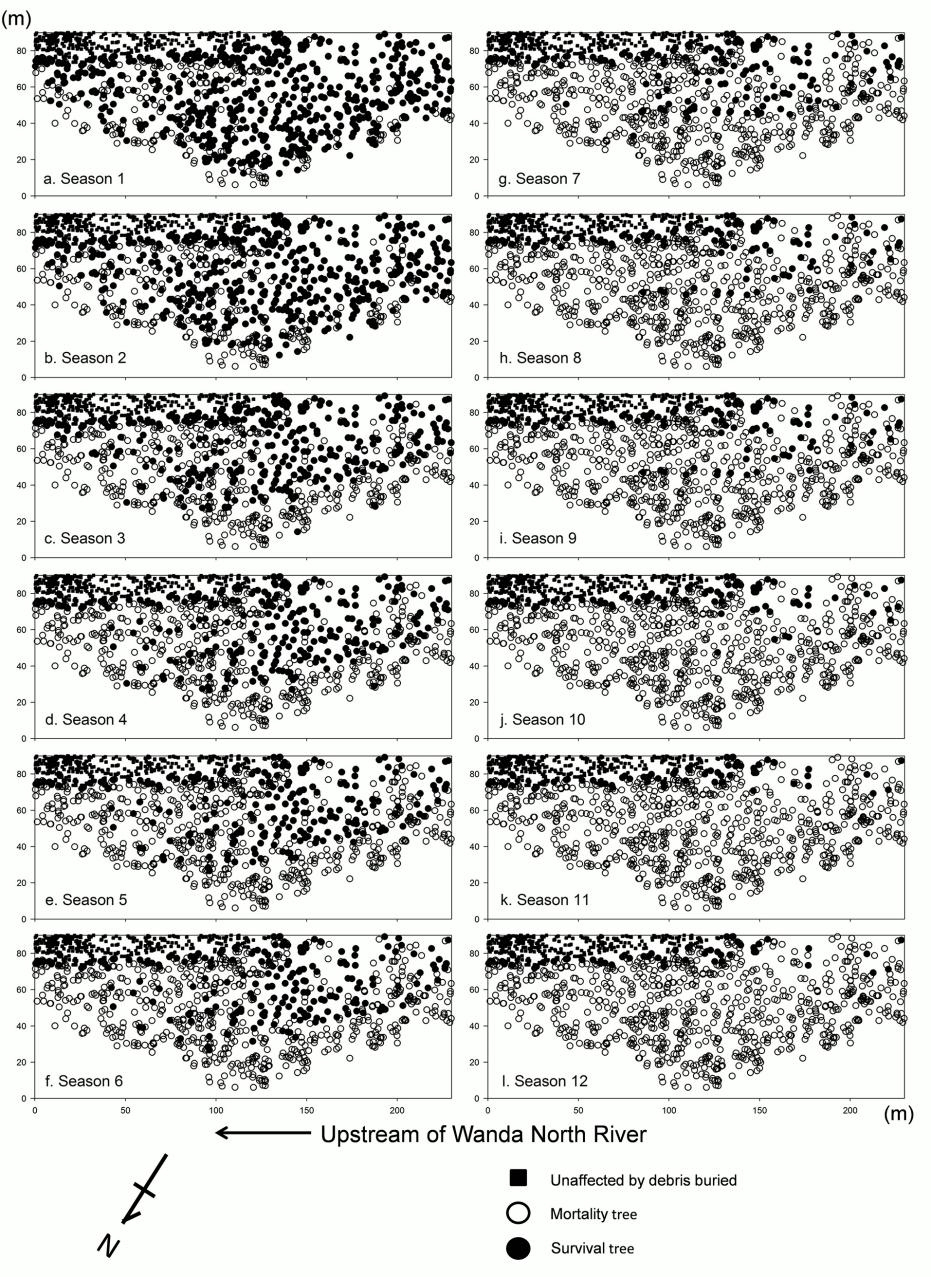
Location of seasonal withered and surviving tree individuals after disturbances by floods and mudslides in the monitored sample plot during 2009–2011.

The stand structure of our long-term monitored plot showed that among the main tree species, the size class distribution of *Liq*. *formosana* was bell shaped (Fig 5A), whereas that of the other five tree species was reverse-J shaped (Fig 5B–5F). Trees distributed upstream and in the margin of forest along the riverbank, which withered and died earlier than other individuals (Figs 6 and 7).

### Tree survival after typhoon–induced floods and mudslides

Our investigation evidenced that 799 trees were affected by floods and mudslides within the monitored sample plot of the Formosan gum forest in December 2008. After Typhoon Morakot in 2009, the size of the area affected by floods increased, and the number of affected trees increased to 856; because the naturally recruited trees were on a slope at a higher elevation above sea level, they were affected to a lesser degree. The five most affected tree species were *Liq*. *formosana* (241 trees), *Z*. *serrata* (142 trees), *Ci*. *insularimontanum* (138 trees), *Cy*. *glauca* (51 trees), and *Sc*. *octophylla* (47 trees), constituting approximately 72.3% of the total number of affected trees, with *Liq*. *formosana* accounting for approximately 28.2% (Table 1).

**Table 1:** Seasonal mortality rates of major tree species after the disturbances by floods and mudslides in the monitored sample Aowanda Formosan gum forest plot during 2009–2011.

Species	Number of debris buried trees	Mortality number / Mortality rate																							
		season 1^a^		season 2		season 3		season 4		season 5		season 6		season 7		season 8		season 9		season 10		season 11		season 12	
		n	(%%)	n	(%%)	n	(%%)	n	(%%)	n	(%%)	N	(%%)	n	(%%)	n	(%%)	n	(%%)	n	(%%)	n	(%%)	n	(%%)
*Liquidambar formosana*	241(235/6)^b^	10	4.1	12	5.0	34	14.1	48	19.9	65	27.0	81	33.6	141	58.5	180	74.7	180	74.7	204	84.7	220	91.3	223	92.5
*Zelkova serrata*	142(137/5)	29	20.4	54	38.0	114	80.3	128	90.1	132	93.0	134	94.4	135	95.1	135	95.1	135	95.1	135	95.1	135	95.1	135	95.1
*Cinnamomum insularimontanum*	138(132/6)	36	26.1	70	50.7	114	82.6	123	89.1	129	93.5	132	95.7	133	96.4	133	96.4	133	96.4	133	96.4	133	96.4	136	98.6
*Cyclobalanopsis glauca*	51(43/8)	24	47.1	26	51.0	34	66.7	44	86.3	49	96.1	49	96.1	49	96.1	49	96.1	49	96.1	49	96.1	50	98.0	50	98.0
*Schefflera octophylla*	47(46/1)	34	72.3	40	85.1	41	87.2	42	89.4	42	89.4	42	89.4	42	89.4	42	89.4	42	89.4	42	89.4	42	89.4	43	91.5
*Carpinus kawakamii*	27(27/0)	10	37.0	17	63.0	23	85.2	27	100.0	27	100.0	27	100.0	27	100.0	27	100.0	27	100.0	27	100.0	27	100.0	27	100.0
*Celtis formosana*	19(15/4)	13	68.4	13	68.4	14	73.7	18	94.7	18	94.7	18	94.7	18	94.7	18	94.7	18	94.7	18	94.7	18	94.7	18	94.7
*Machilus zuihensis*	16(16/0)	8	50.0	11	68.8	13	81.3	16	100.0	16	100.0	16	100.0	16	100.0	16	100.0	16	100.0	16	100.0	16	100.0	16	100.0
*Glochidion rubrum*	15(13/2)	1	6.7	2	13.3	8	53.3	11	73.3	12	80.0	12	80.0	12	80.0	13	86.7	13	86.7	13	86.7	13	86.7	14	93.3
*Quercus variabilis*	14(13/1)	6	42.9	6	42.9	7	50.0	11	78.6	11	78.6	11	78.6	12	85.7	12	85.7	12	85.7	12	85.7	12	85.7	12	85.7
*Swida macrophylla*	13(13/0)	4	30.8	7	53.8	11	84.6	12	92.3	13	100.0	13	100.0	13	100.0	13	100.0	13	100.0	13	100.0	13	100.0	13	100.0
*Oreocnide pedunculata*	12(7/5)	4	33.3	5	41.7	5	41.7	11	91.7	11	91.7	11	91.7	11	91.7	11	91.7	11	91.7	11	91.7	11	91.7	11	91.7
*Largerstoemia subcostata*	9(9/0)	0	0.0	0	0.0	1	11.1	1	11.1	5	55.6	5	55.6	5	55.6	5	55.6	5	55.6	5	55.6	5	55.6	7	77.8
*Murraya euchrestifolia*	9(7/2)	2	22.2	2	22.2	2	22.2	4	44.4	4	44.4	4	44.4	4	44.4	4	44.4	4	44.4	4	44.4	5	55.6	5	55.6
*Syzygium formosanum*	9(6/3)	1	11.1	2	22.2	3	33.3	7	77.8	7	77.8	7	77.8	7	77.8	7	77.8	7	77.8	8	88.9	8	88.9	8	88.9
*Belischmiedia erythrophloia*	9(8/1)	2	22.2	4	44.4	5	55.6	6	66.7	7	77.8	7	77.8	7	77.8	7	77.8	7	77.8	7	77.8	7	77.8	7	77.8
*Idesia polycarpa*	5(5/0)	4	80.0	4	80.0	5	100.0	5	100.0	5	100.0	5	100.0	5	100.0	5	100.0	5	100.0	5	100.0	5	100.0	5	100.0
*Prunus campanulata*	5(5/0)	3	60.0	5	100.0	5	100.0	5	100.0	5	100.0	5	100.0	5	100.0	5	100.0	5	100.0	5	100.0	5	100.0	5	100.0
*Elaeocarpus sylvestris*	5(4/1)	4	80.0	4	80.0	4	80.0	5	100.0	5	100.0	5	100.0	5	100.0	5	100.0	5	100.0	5	100.0	5	100.0	5	100.0
Others ^c^	70(58/12)	30	42.9	38	54.3	46	65.7	58	82.9	61	87.1	61	87.1	61	87.1	61	87.1	61	87.1	61	87.1	61	87.1	64	91.4
Total	856(799/57)	225	26.3	322	37.6	489	57.1	582	68.0	624	72.9	645	75.4	708	82.7	748	87.4	748	87.4	773	90.3	791	92.4	804	93.9

^a^The records were denoted in 2009 as season 1 (January), 2 (March), 3 (August), and 4 (November); in 2010 as seasons 5 (January), 6 (March), 7 (July), and 8 (October); and in 2011 as season 9 (January), 10 (April), 11 (August), and 12 (October).

^b^Data in the parentheses indicate “number of trees affected by floods/number of trees unaffected by floods.”

^c^Tree species with fewer than five individuals in the sample area were combined.

**Fig 8:**
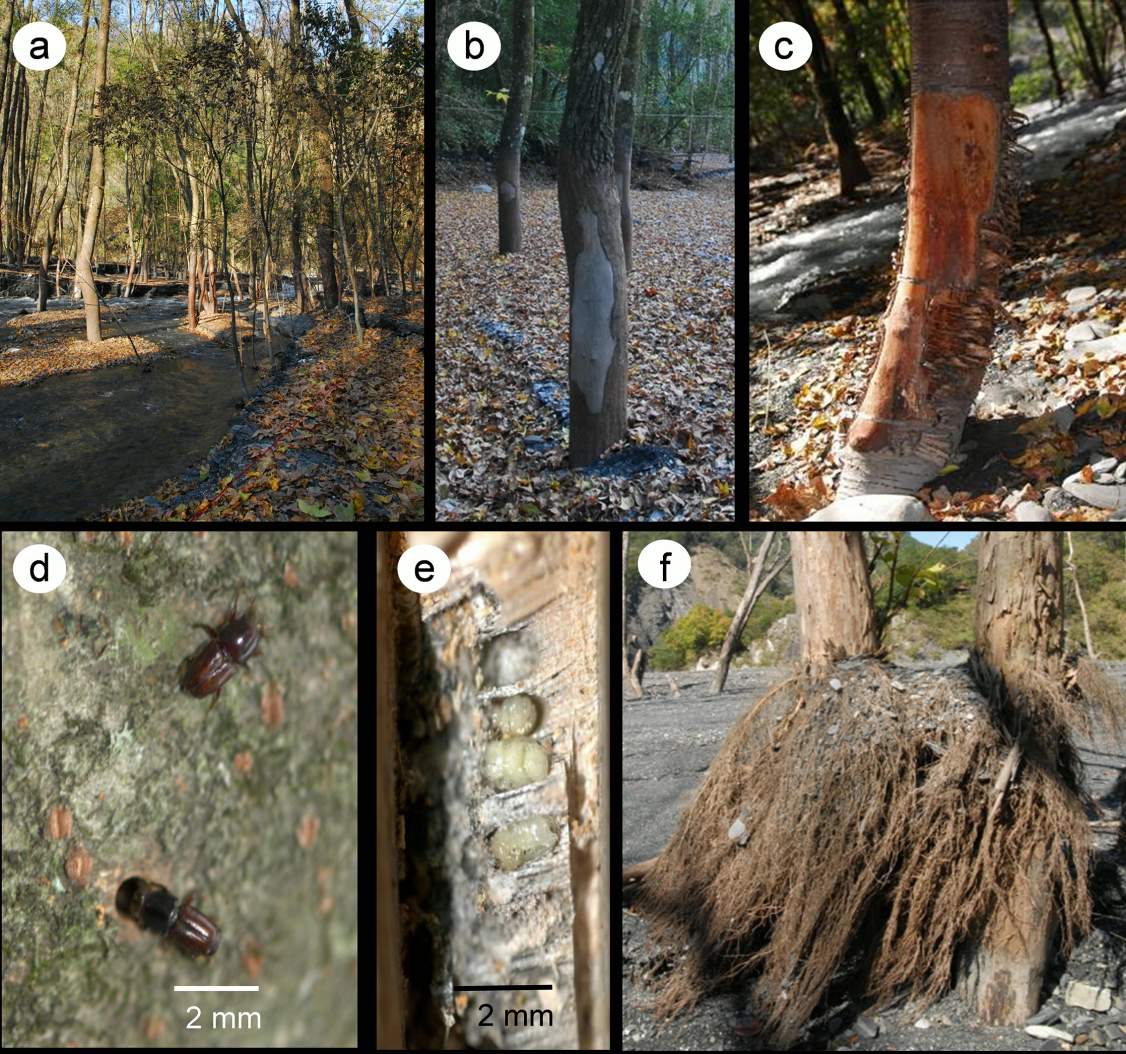
Conditions after disturbances by floods and mudslides in the monitored sample plot. (a) The river changed courses, and sand and gravel piled up. (b–c) Peeled bark of *Liquidambar formosana* (b) and *Prunus campanulata* (c) after sand and gravel impact. (d–e) Trees harboring bark beetles. (f) Rerooting of *Lagerstoemia subcostata* after floods.

After disturbances by Typhoon Sinlaku–induced floods and mudslides, our season 1 survey revealed that 799 trees were washed out by the north Wanda River and were impacted by the piles of sand and gravel in the deposited areas. Among these, 225 trees withered (season 1 withering rate, 28.2%). The sand and gravel impacted and deposition directly damaged trees to a severe extent (Fig 8A–8C). Some tree species including *Prunus campanulata*, *Swida macrophylla*, and *Sc*. *octophylla* and those with small DBH died in season 1 because of the peeling of their bark caused by the sand and gravel impact. The season 2 survey displayed that 97 of 574 surviving trees incrementally withered (season 2 withering rate, 16.9%). According to the season 3 survey, 167 of 477 surviving trees had withered (season 3 withering rate, 35.0%).

**Fig 9:**
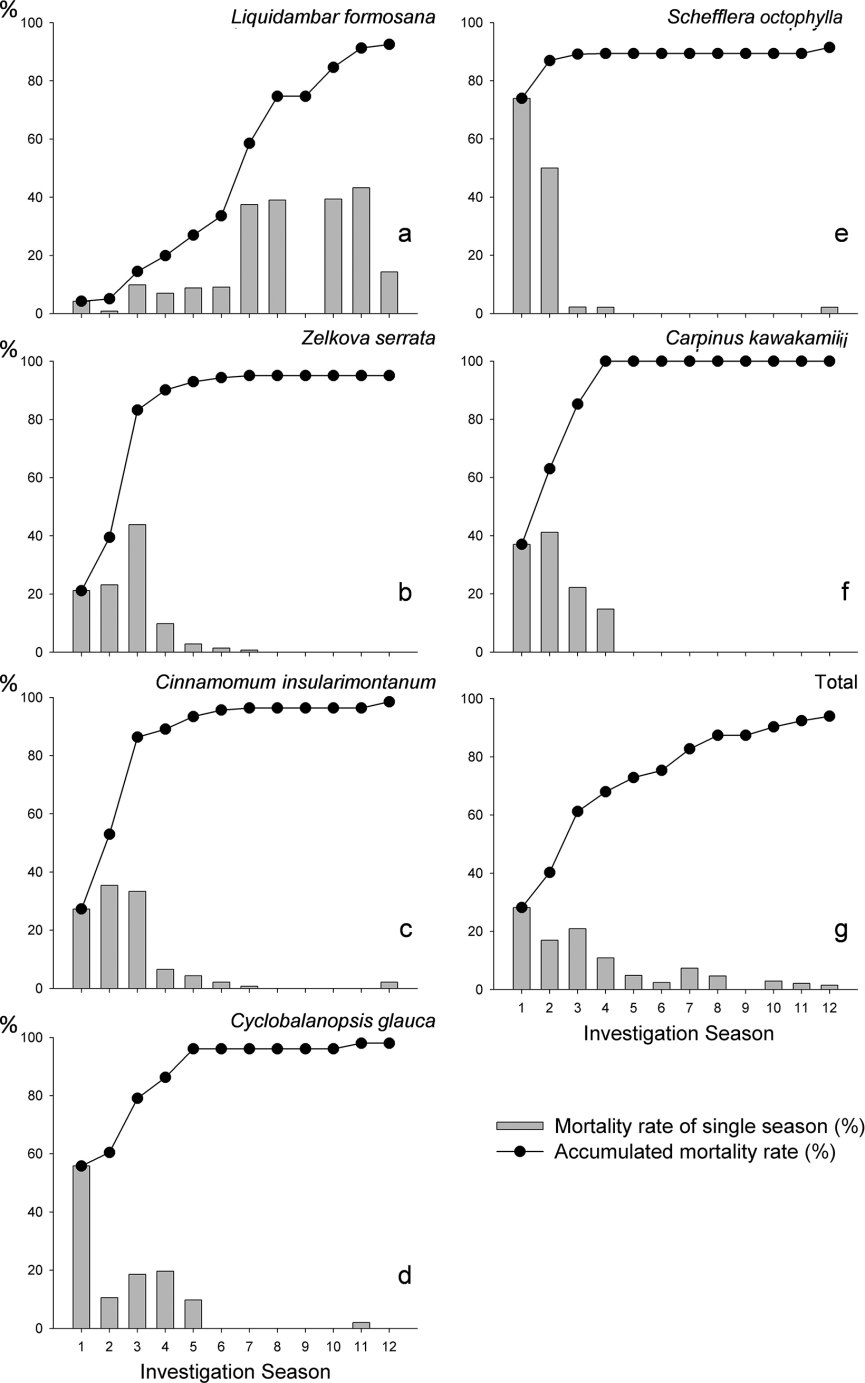
Seasonal and accumulated mortality rates of the major tree species after disturbances by floods and mudslides in the longitudinally monitored sample Aowanda Formosan gum forest plot during 2009–2011.

*Ci*. *insularimontanum* and *Z*. *serrata* were the species that mainly withered during the study period of season 3 survey (Fig 9). Because Typhoon Morakot affected Taiwan during August 7–9, 2009, sand and gravel piles were >3 m higher than the height of the pile recorded in the Formosan gum forest during season 4, increasing the area impacted by sand and gravel in the Formosan gum forest. An additional 57 trees were affected by mudslides, most of which were young shade-tolerant trees species such as *Oreocnide pedunculata*, *Murraya euchrestifolia*, *Litsea elongata*, *Cy*. *glauca*, and *Z*. *serrata* [11]. Among the trees affected by newly deposited sand and gravel in season 4, the withering rate of species including *Lit*. *elongata*, *O*. *pedunculata*, *M*. *euchrestifolia*, *Syzygium formosanum*, and *Celtis formosana* was >50%. Ninety-three trees withered in season 4 (withering rate, 25.3%). Therefore, in total, 582 trees withered in the first year (cumulative withering rate, 68.0%; Table 1).

During the season 5 survey in 2010 (second year), 232 of 856 trees survived on the long-term monitored plot in the Formosan gum forest (season 5 withering rate, 15.3%); only three tree species had a survival rate of >30%: *Liq*. *formosana* (73.0%), *M*. *euchrestifolia* (55.6%), and *Lagerstoemia subcostata* (44.4%). The most stems of *La*. *subcostata* had begun rooting after the disturbances (Fig 8F). After Typhoon Morakot, the pile of sand and gravel increased by 2–3 m. Tree species such as *Carpinus kawakamii*, *Machilus zuihoensis*, *Sw*. *macrophylla*, *Idesia polycarpa*, *Pr*. *campanulata*, and *Elaeocarpus sylvestris* had died. In season 6, only 211 trees survived (cumulative withering rate, 75.4%). In season 7, the mortality rate of trees was >80% and 63 trees died (withering rate, 29.9%). The season 8 survey showed that only 108 of 856 trees survived (cumulative withering rate, 87.4%).

The results from the season 9 survey in 2011 (third year) were the same as those of the season 8. Only 83 trees had survived by season 10 (survival rate, 9.7%; withering rate, >90%; Table 1). Of the 25 trees that died in season 10, 24 were of *Liq*. *formosana* and one of *S*. *formosanum*. In season 11, only 64 trees survived (survival rate, 7.5%). Thus far, the cumulative number of withered trees was 792 (cumulative withering rate, 92.5%). Of the 19 trees withering during season 11, 16 were of *Liq*. *formosana* and 3 were of *Cy*. *glauca*, *Z*. *serrata*, and *M*. *euchrestifolia* each. In season 12, only 52 trees survived (mortality rate, 93.9%).

To understand the effects of sand and gravel and floods on tree growth and survival in the Formosan gum forest, we compared the survival rate of different tree species. Results revealed that the length of survival for the six major species in the long-term monitored plot varied significantly (p < 0.05). Using multiple comparisons (Table 2), the lifespan of *Liq*. *formosana* was found to be significantly longer than that for the other five species. The lifespans of *Z*. *serrata*, *Ci*. *insularimontanum*, *Cy*. *glauca*, and *Ca*. *kawakamii* were similar. However, the lifespan of *Sc*. *octophylla* was significantly shorter.

**Table 2:** Multiple regression analyses and comparison of the average lifespans (in months), relative positions, and DBHs of the major tree species after disturbances by floods and mudslides in the monitored sample Aowanda Formosan gum forest plot during 2009–2011.

Species	n	Mean(±SE)^a^			Standardized β-value			R^2^
					DU^b^	DS^c^	DBH^d^	
*Liquidambar formosana*	241	22.3	±	7.3^A^	**0.12**^e^	-**0.26**	**0.46**	**0.34**
*Zelkova serrata*	142	10.2	±	3.8^B^	0.14	-**0.26**	**0.29**	**0.18**
*Cinnamomum insularimontanum*	138	6.9	±	3.2^B^	**0.14**	0.21	**0.14**	**0.08**
*Cyclobalanopsis glauca*	51	9.0	±	6.4^B^	0.03	-**0.56**	0.18	**0.31**
*Schefflera octophylla*	47	5.3	±	5.3^C^	**-**0.13	-**0.47**	**0.62**	**0.46**
*Carpinus kawakamii*	27	7.4	±	3.8^B^	**-**	**-**	**-**	**-**

^a^Means followed by the same letter are not significantly different at p < 0.05.

^b^DU: distance from upstream.

^c^DS: distance from sampled tree to the above boundary of sample plot.

^d^DBH: diameter at breast height.

p values < 0.05 are given in bold.

The seasonal and accumulated mortality rates indicated a difference in adaptability of tree species under the disturbances by floods and mudslides (Table 1 and Fig 9). Based on mortality rates of flood disturbance, tree species could be classified into three categories as following: tolerant, moderately tolerant, and intolerant forms. The tolerant form, including *Liq*. *formosana* (Fig 9A) and *La*. *subcostata*, was the most resistant to the effect of floods and mudslides. The moderately tolerant form, including *Z*. *serrata* (Fig 9B), *Ci*. *insularimontanum* (Fig 9C), *Cy*. *glauca* (Fig 9D), and *Ca*. *kawakamii* (Fig 9F), exhibited a moderate adaptability to flood disturbances. In season 3, the accumulated mortality rates were >80%, but the mortality rate trend varied slightly among different tree species. Of the species belonging to the intolerant form, only *Sc*. *octophylla* (Fig 9E) had a mortality rate close to 80% in season 1 after disturbances by floods, demonstrating that this species had the weakest flood resistance. With fewer trees in the sample area, *Pr*. *campanulata* and *Sw*. *macrophylla* were also categorized under the intolerant form.

After our 3-year monitoring, we observed that the area with withered trees within the sand- and gravel- deposited areas gradually began increasing, and with time, the number of trees dying after the disturbances also increased (Figs 7 and 9). If trees in the sample area began growing weakly in the first year or their leaves began wilting in summer, they typically died in spring or summer of the second year. Some trees with smaller DBH located near the river channel generally withered early. The trend of the location where trees withered in the sample area started from upstream of the north Wanda River and locations closer to the river channel (farther from slopes) and gradually stretched downstream of the north Wanda River and locations farther away from the course of the river (closer to slopes; Fig 7).

Multiple regression analysis was performed for the lifespans of five major tree species at a distance from the upstream of the river, at a distance from the riverbank, and DBH (Table 2). DBH and lifespan displayed significantly positive correlation with each other, whereas the distance from the riverbank and lifespan showed significantly negative correlation. Only the lifespans of *Liq*. *formosana* and *Ci*. *insularimontanum* were significantly correlated with the distance from upstream. On comparing the standardized beta values for *Liq*. *formosana*, the positive effect of DBH on lifespan (0.46) was greater than that of the distance from the riverbank (-0.26) or distance from upstream (0.12). For *Z*. *serrata*, the effects of DBH and the distance from the riverbank on lifespan were similar (0.29 and -0.26, respectively), but the distance from upstream had no significant effect. Although the *Ci*. *insularimontanum* model was statistically significant, its degree of explanation was slightly low (R^2^ = 0.08). For *Cy*. *glauca*, only distance from the riverbank revealed a significant correlation with lifespan (-0.56). The results for *Sc*. *octophylla* indicated that the positive effect of DBH (0.62) was greater than the negative effect of the distance from the riverbank (-0.47) on lifespan. Thus, the larger the DBH, the shorter the distance from the riverbank, and the longer the distance from the upstream, the longer was the lifespan. However, none of our tree species models completely explained these results (R^2^ = 0.08–0.46).

We also observed that in addition to tree species, DBH, and position, insect infestation was a major factor determining the survival rate of trees. Most tree species were affected by floods and mudslides such as *Z*. *serrata*, *Ci*. *insularimontanum*, and *Ca*. *kawakamii*, particularly those located at the margin of the Formosan gum forest along the river side, died between seasons 2 and 3; nevertheless, some of these tree species survived in season 1. During season 1, the dung of bark beetles (Scolytidae, Scolytinae, *Scolytoplatypus raja*) was noted on the trunks of the aforementioned trees (Fig 8D and 8E). Among the trees with bark beetle infestation, most died in season 2 (Fig 9). Furthermore, our season 3 survey (August 2009) showed that the trunk bases of flood- and mudslide-affected *Liq*. *formosana* harbored bark beetles. In the second year, *Liq*. *formosana* started dying (Table 1 and Fig 9).

## Discussion

### Vegetation structure

The locations, geographical features, and compositions of riparian vegetation are relatively affected by variations in intensity, frequency, and duration of water flow [17, 26]. In addition, riparian vegetation is affected by the processes and interactions of regional weather, geological structure, and biology; composition, structure, and productivity of the vegetation are correlated with topography, geomorphology, sand, water, disturbances, and river terraces, which lead to its mosaic or zonated distribution [17, 27, 28, 29, 30]. As such, sand and gravel have piled up and formed a terrace along the north and south Wanda River over a long period. This has decreased disturbances by the river water and led to plant invasion; thus, a riparian forest has formed. This has gradually resulted in the current plant distribution in the Aowanda Formosan gum forest (Fig 4).

In our long-term monitored sample plot, the main tree species include *Liq*. *formosana*, *Z*. *serrata*, *Ci*. *insularimontanum*, *Cy*. *glauca*, and *Sc*. *octophylla*; these species constitute riparian vegetation belonging to *Liq*. *formosana* subtype, *Ci*. *insularimontanum* type along the Wanda River riverbank [22]. According to our results, the Formosan gum forest could be classified further into four types, which expressed a characteristic distribution for each dominant tree species ecological niche within the forest in a smaller spatial scale. Moderately shade-tolerant trees such as *Sc*. *octophylla* [31] were abundantly distributed upstream of the river, where the terrace was higher and the sky light space was smaller than the terrace at lower reaches of the river. However, most individuals of another moderately shade-tolerant species, *Ci*. *insularimontanum* [31] were concentrated under the crowns of *Liq*. *formosana* and *Z*. *serrata* along the margin of the plot in the downstream. Most individuals of pioneer tree species *Liq*. *formosana* [31], were majorly distributed downstream with a larger sky light space. Furthermore, another pioneer tree species *Z*. *serrata* [31], was distributed widely within the sample area.

Moreover, the distribution of the size classes of the dominant tree species within the Formosan gum forest could roughly be classified into two types: bell shaped for *Liq*. *formosana* and reverse-J shaped for *Z*. *serrata*, *Ci*. *insularimontanum*, *Cy*. *glauca*, *Sc*. *octophylla*, and *Ca*. *kawakamii*. These results were generally similar to those of the Wanda River riparian vegetation study by Chung [22]; the author observed that although it was a dominant tree species in *Liq*. *formosana* subtype, *Ci*. *insularimontanum* type community, young trees of *Liq*. *formosana* were fewer in number, and that if the riparian vegetation remained undisturbed, it was replaced by a shade-tolerant tree species such as *Sc*. *octophylla*.

### Tree survival after typhoon-induced floods and mudslides

On comparing the tree distribution patterns in the study area following disturbances by floods and mudslides, Chung [22] reported that although *Z*. *serrata*, *Ci*. *insularimontanum*, *Cy*. *glauca*, *Sc*. *octophylla*, and *Ca*. *kawakamii* were often present in the Wanda riparian vegetation, they also had a greater distribution in the higher river terrace. Furthermore, these tree species appeared among other riparian vegetation in Taiwan [12, 13, 32]. However, these trees had poorer tolerance for disturbances by mudslides and a higher mortality rate (approximately 90%) in the period immediately after the disturbances compared with *Liq*. *formosana* and *La*. *subcostata*. The riparian vegetation species varied because of differences in altitude, topography, location, and habitat and endured different levels of intensity and frequency of disturbances by floods and mudslides. Furthermore, every species had varied adaptability strategies in the river environment, and this was reflected in the differences in their means of growth and survival after disturbances [12, 17, 18, 19, 29, 33]. Based on our 3-year monitoring and analyses, survival rates evidenced significant different among some tree species after floods and mudslides.

During their growth season (i.e., spring and summer), plants have a lower tolerance for disturbances (stress), thus exhibiting a higher mortality rate; however, because of dormancy during fall and winter, plant tolerance to disturbances increases [34, 35]. In addition to causing direct physical damage, they also prevented seeds from germinating, individuals from growing, and propagules from developing, further causing early aging or death of plants [17, 36]. When the Formosan gum forest was submerged in water and then buried under and impacted by sand and gravel in mid-September 2008, deciduous trees such as *Liq*. *formosana*, *Z*. *serrata*, and *Ca*. *kawakamii* started entering dormancy. Their leaf phenology started changing from yellowing to defoliation, and their physiological function became weaker than that during the growth season. Therefore, the tree mortality rate was lower during the initial period of disturbances by floods and mudslides. Although very few studies have reported on *Liq*. *formosana* as a dominant species in the riparian vegetation in Taiwan, a congener *Liq*. *styraciflua* has been shown to have a favorable tolerance to encroachment by floods [37, 38, 39, 40, 41, 42]. Pezeshki and Chamber [40] showed that the sprouts of *Liq*. *styraciflua* can increase their flood tolerance through rapid recovery of photosynthesis and pores during short-term floods. In their 2-year flood simulation study, Angelov et al. [41] revealed that the mortality rate of *Liq*. *styraciflua* was <5%, indicating that it has favorable adaptability to conditions such as flooding or submersion of roots.

During floods, tree species tolerant to submergence typically show increased trunk size, enlarged pores, adventitious roots, delayed aging of leaves at the trunk base, increased alcohol dehydrogenase and superoxide dismutase activities and adaptability to anaerobic environments, small decreases in pore conductivity and net photosynthesis rates, and faster recovery [36, 43, 44]. In general, trees intolerant to submergence do not have the aforementioned characteristics. Our 3-year observation showed that some tree species such as *La*. *subcostata* (Fig 8F), *Psychotria rubra*, and *Morus australis* had adventitious roots; except for *La*. *subcostata*, these tree species were mainly distributed closer to slopes and away from river channels. Even after minor impacts of mudslides, trees with adventitious roots can survive. *La*. *subcostata* shows highly favorable rooting along with adventitious roots; therefore, it is mainly used for vegetation engineering in the collapsed earthen slope areas of Taiwan [45]. The Aowanda Formosan gum forest has sand and gravel deposition and unstable rivers; the adventitious roots of the aforementioned trees become exposed after the river washed out sand and gravel, thus causing additional damage and increasing tree mortality rates.

The differences in both adaptability and DBH of trees were affected their resistance to adversities such as disturbances by floods [17, 46]. The roots of large trees with large DBH spread deeply and widely for relatively more favorable physiological conditions, fixing capability, and physical damage resistance during adversities [17, 18, 36, 46, 47]. Finally, the results of the present study also revealed that the larger the DBH of trees, the higher is their resistance to disturbances by floods.

The results of this study displayed that trees distributed at the margin of the Formosan gum forest (closer to the river) die faster after floods than those located far from the river. The distance of trees from rivers determines the strength of the disturbances; therefore, the nearer the river and the stronger flow speed and disturbances, the higher is the tree mortality rate [17, 18, 19, 29, 48]. Because environmental changes are rapid at the margin of the Formosan gum forest close to the river (eg. the temperature of the river gravel bed under the margin of forest can be >65°C in summer, and the humidity at the margin of the forest can change severely more than within forest between day and night), the wounded trees are further damaged, resulting in their difficulty in surviving. Furthermore, according to the position of trees withering and surviving on our long-term monitored sample plot during the 3-year survey period (Figs 6 and 7), trees upstream along the river died soon after the disturbances; this might indicate that trees in a downstream position had a higher survival rate because of protection provided by trees upstream. Therefore, the relative position of trees in the riparian zone affects their survival rate after disturbances.

We observed that before withering, most trees were infested by bark beetles after floods and mudslides. Although *Liq*. *formosana* had a stronger tolerance to floods and mudslides, it was also affected by bark beetles after the disturbances occurring approximately two to three seasons later. Bark beetles are one of the major forest pests [49, 50]. Disturbances by factors, such as fire, floods, or drought, reduce the physiological and biochemical activities of trees, easing invasion by bark beetles, finally resulting in tree death [51, 52, 53, 54]. Healthy trees, which have not been disturbed by floods and mudslides, remain bark beetle-free. This difference could be because volatile odors (such as α-pinene and ethanol), which facilitate bark beetles in finding suitable hosts, vary between healthy and wounded trees [55, 56]. Resins are attributable to resistance of healthy trees to bark beetles; however, in trees with peeled bark or in physically weak trees, the resin levels are lower, leading to lower resistance to bark beetles, easing invasion by the beetles and tree death through fungal infection [49].

The Aowanda Formosan gum forest area, the most crucial natural landscape in the Aowanda National Forest Recreation Area in Taiwan, contains unique riparian vegetation, mainly dominated by *Liq*. *formosana*. Most trees are damaged by floods, and they die consequently because of bark beetle invasion; however, our study area is located at the watershed area in the Wanda reservoir where insect infestation is an ecological incident caused by special weather and geographical conditions. To ensure residential water safety and preserve natural ecology, pesticide use, for preventing bark beetle invasion, is prohibited.

Our 3-year survey of the Aowanda Formosan gum forest area showed that factors such as differences in tree species, DBH, and relative position affect tree survival rates after disturbances by floods and mudslides. Some preceding factors are correlated; for example, tree species and DBH are correlated: the dominant tree species *Liq*. *formosana* and *Z*. *serrata* had the largest average DBHs. The relative location of trees was also correlated with tree species: most *Liq*. *formosana* trees were located downstream, *Sc*. *octophylla* and *Cy*. *glauca* upstream, and *Ci*. *insularimontanum* and *Z*. *serrata* in areas proximal to the river. Our study revealed that the effect of disturbances on tree survival in the Aowanda Formosan gum forest area is multifactorial.

Typhoons are major factors in subtropical ecosystems of Asia-Pacific. In Formosan gum forest area, severe typhoon-induced floods and mudslides interfered with the riparian vegetation, replacing the original vegetation and beginning secondary succession; nevertheless, this provided a new habitat to plant propagules, enabling them to form their individual colonies. Their seedlings were established in the second year after the disturbances occurred (data not shown). Hence, we could understand the lifecycle of riparian vegetation after disturbances by typhoon-induced floods and mudslides, from destruction to re-establishment. Extreme climatic conditions have accelerated the frequency of intense weather incidents; thus, further research concerning the impact of disturbances on the succession process of riparian vegetation in subtropical areas is required.

## Conclusions

The relative location where main tree species withered after disturbances by floods and mudslides tended to be on the river side of the edge of Aowanda Formosan gum forest area, rather than relatively farther from the river. This showed that trees distributed along the outer rim of the area were relatively more threatened by mudslides and withered more easily. The mortality rates of the major tree species varied significantly depending on the season; the mortality rate was higher in summer and fall. The DBHs of withered and surviving trees varied significantly throughout the survey period. The larger the DBH of a tree, the higher was its tolerance to flood and mudslide impact and deposition. Tree species had significantly different tolerance to disturbances by floods and mudslide; *Liq*. *formosana* and *La*. *subcostata* had the most favorable tolerance. Thus, these tree species can be used for vegetation engineering in areas with frequent floods at low to medium elevation in Taiwan. After disturbances, tree species are prone to secondary damage by bark beetle infestation and fungal infection. Most trees with this secondary damage died in our study. Bark beetles were found to be euryphagous insects, which invade trees only under poor physiological conditions.

The inundation and deposition by floods and mudslides in the Aowanda Formosan gum forest is a distinct ecological incident resulting from the interaction between geology and weather. First, earthquakes loosen the sand and gravel on mountain sides; extremely heavy rains then cause mudslides, leading to floods and mudslides that impact the particular area. The changes in topography and geomorphology affect existing as well as future riparian vegetation. Studies focusing on the survival rate of riparian vegetation after floods and mudslides occur are scant. Therefore, the current study could provide basic information regarding ecological management of riparian vegetation in subtropical areas under extreme climatic changes.

## Supporting information

S1 Dataset The raw data of this study uploaded as “S1_Dataset.xlsx” file. (XLSX)
